# The clinical and genetic heterogeneity of paroxysmal dyskinesias

**DOI:** 10.1093/brain/awv310

**Published:** 2015-11-18

**Authors:** Alice R. Gardiner, Fatima Jaffer, Russell C. Dale, Robyn Labrum, Roberto Erro, Esther Meyer, Georgia Xiromerisiou, Maria Stamelou, Matthew Walker, Dimitri Kullmann, Tom Warner, Paul Jarman, Mike Hanna, Manju A. Kurian, Kailash P. Bhatia, Henry Houlden

**Affiliations:** 1 MRC Centre for Neuromuscular Diseases, UCL Institute of Neurology, Queen Square, London WC1N 3BG, UK; 2 Department of Molecular Neuroscience, UCL Institute of Neurology, Queen Square, London WC1N 3BG, UK; 3 Paediatrics and Child Health, Children’s Hospital, Westmead, University of Sydney, Australia; 4 Neurogenetics Laboratory, UCL Institute of Neurology, Queen Square, London WC1N 3BG, UK; 5 Department of Motor Neuroscience and Movement Disorders, UCL Institute of Neurology, Queen Square, London WC1N 3BG, UK; 6 Developmental Neurosciences, UCL Institute of Child Health, London WC1N 3JH, UK; 7 Department of Neurology, Papageorgiou Hospital, Thessaloniki University of Athens, Greece; 8 Department of Neurology University of Athens, Greece; 9 Department of Neurology, Philipps University, Marburg, Germany; 10 Department of Experimental Epilepsy, UCL Institute of Neurology, Queen Square, London WC1N 3BG, UK; 11 Department of Neurology, Great Ormond Street Hospital, London WC1N, UK

**Keywords:** PRRT2, SLC2A1, PNKD, gene, paroxysmal movement disorder

## Abstract

Paroxysmal dyskinesia can be subdivided into three clinical syndromes: paroxysmal kinesigenic dyskinesia or choreoathetosis, paroxysmal exercise-induced dyskinesia, and paroxysmal non-kinesigenic dyskinesia. Each subtype is associated with the known causative genes *PRRT2*, *SLC2A1* and *PNKD*, respectively. Although separate screening studies have been carried out on each of the paroxysmal dyskinesia genes, to date there has been no large study across all genes in these disorders and little is known about the pathogenic mechanisms. We analysed all three genes (the whole coding regions of *SLC2A1* and *PRRT2* and exons one and two of *PNKD*) in a series of 145 families with paroxysmal dyskinesias as well as in a series of 53 patients with familial episodic ataxia and hemiplegic migraine to investigate the mutation frequency and type and the genetic and phenotypic spectrum. We examined the mRNA expression in brain regions to investigate how selective vulnerability could help explain the phenotypes and analysed the effect of mutations on patient-derived mRNA. Mutations in the *PRRT2*, *SLC2A1* and *PNKD* genes were identified in 72 families in the entire study. In patients with paroxysmal movement disorders 68 families had mutations (47%) out of 145 patients. *PRRT2* mutations were identified in 35% of patients, *SLC2A1* mutations in 10%, *PNKD* in 2%. Two PRRT2 mutations were in familial hemiplegic migraine or episodic ataxia, one *SLC2A1* family had episodic ataxia and one *PNKD* family had familial hemiplegic migraine alone. Several previously unreported mutations were identified. The phenotypes associated with *PRRT2* mutations included a high frequency of migraine and hemiplegic migraine. *SLC2A1* mutations were associated with variable phenotypes including paroxysmal kinesigenic dyskinesia, paroxysmal non-kinesigenic dyskinesia, episodic ataxia and myotonia and we identified a novel *PNKD* gene deletion in familial hemiplegic migraine. We found that some *PRRT2* loss-of-function mutations cause nonsense mediated decay, except when in the last exon, whereas missense mutations do not affect mRNA. In the *PNKD* family with a novel deletion, mRNA was truncated losing the C-terminus of PNKD-L and still likely loss-of-function, leading to a reduction of the inhibition of exocytosis, and similar to PRRT2, an increase in vesicle release. This study highlights the frequency, novel mutations and clinical and molecular spectrum of *PRRT2*, *SLC2A1* and *PNKD* mutations as well as the phenotype–genotype overlap among these paroxysmal movement disorders. The investigation of paroxysmal movement disorders should always include the analysis of all three genes, but around half of our paroxysmal series remain genetically undefined implying that additional genes are yet to be identified.

## Abstract (precis)

The contributions of different genes to inherited paroxysmal movement disorders are incompletely understood. Gardiner *et al*. identify mutations in 47% of 145 individuals with paroxysmal dyskinesias, with PRRT2 mutations in 35%, SLC2A1 in 10% and PNKD in 2%. New mutations expand the associated phenotypes and implicate overlapping mechanisms.

## Abstract (precis)

The contributions of different genes to inherited paroxysmal movement disorders are incompletely understood. Gardiner *et al.* identify mutations in 47% of 145 individuals with paroxysmal dyskinesias, with PRRT2 mutations in 35%, SLC2A1 in 10% and PNKD in 2%. New mutations expand the associated phenotypes and implicate overlapping mechanisms.

## Graphical abstract

**Figure:**
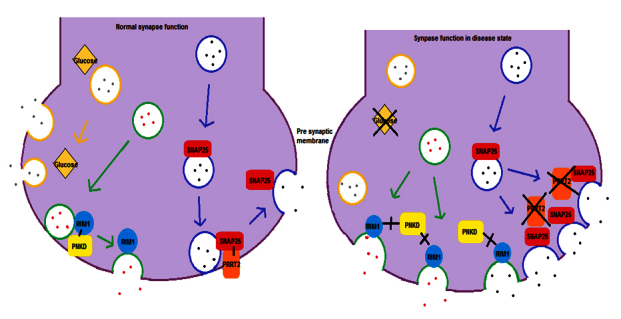
The contributions of different genes to inherited paroxysmal movement disorders are incompletely understood. Gardiner *et al*. identify mutations in 47% of 145 individuals with paroxysmal dyskinesias, with PRRT2 mutations in 35%, SLC2A1 in 10% and PNKD in 2%. New mutations expand the associated phenotypes and implicate overlapping mechanisms.

## Introduction

Paroxysmal dyskinesia was first reported in 1892 by Shuzo Kure in a 23-year-old Japanese man, who had frequent movement-induced paroxysmal attacks from the age of 10 years. At that time the diagnosis was referred to as atypical Thomsen’s disease (Kure, 1892). Later, Gowers (1901) described a similar child, but he considered this movement disorder an epileptic phenomenon, and in 1940, Mount and Reback (1940) described a 23-year-old with involuntary writhing and posturing of the trunk and extremities and labelled this condition paroxysmal dystonic choreoathetosis. Kertesz (1967) and Weber (1967) described families with this condition termed paroxysmal kinesigenic choreo-athetosis and familial paroxysmal dystonia, and Demirkiran and Jankovic (1995) amalgamated the many terms used, suggesting three subtypes, comprising paroxysmal kinesigenic (PKD or PKC), non-kinesigenic (PNKD), and exercise-induced dyskinesia (PED) (Bruno *et al.*, 2004, 2007; Bhatia, 2011). A fourth type, paroxysmal hypnogenic dyskinesia (PHD), characterized by attacks of dyskinesia during sleep, was previously included, but has since been recognized as autosomal dominant nocturnal frontal lobe epilepsy (Sohn and Lee, 2011).

The most common of the paroxysmal movement disorders is PKD, in which attacks are precipitated by voluntary movements such as standing from a sitting position, or the transition from walking to running. Onset is usually in childhood, and attacks are often controlled by carbamazepine (Bhatia, 2001, 2011; Erro *et al.*, 2014). PKD is frequently preceded by infantile convulsions, often with choreoathetosis. The gene responsible for PKD proved elusive for many years, but was recently identified as *PRRT2*, which encodes a small proline-rich transmembrane protein (Chen *et al.*, 2011; Wang *et al.*, 2011; Cloarec *et al.*, 2012; de Vries *et al.*, 2012; Gardiner *et al.*, 2012; Guerrini and Mink, 2012; Hedera *et al.*, 2012; Heron *et al.*, 2012; Li *et al.*, 2012; Liu *et al.*, 2012; Scheffer *et al.*, 2012). The function of the protein is unknown, but it has been shown to interact with the synaptic protein SNAP25 (Lee *et al.*, 2012). Mutations in the *PRRT2* gene account for a large proportion of PKD and several groups have reported mutations in this gene (Chen *et al.*, 2011; Wang *et al.*, 2011; Cao *et al.*, 2012; de Vries *et al.*, 2012; Friedman *et al.*, 2012; Gardiner *et al.*, 2012; Heron *et al.*, 2012; Lee *et al.*, 2012; Li *et al.*, 2012; Liu *et al.*, 2012; Ono *et al.*, 2012; Ishii *et al.*, 2013; Specchio *et al.*, 2013).

Attacks of PNKD are usually triggered by alcohol, coffee or strong emotion. They last longer than attacks of PKD, often from 10 min to 1 h, but can last as long as 12 h. However, they are much more infrequent and occur only a few times a year (Mount and Reback, 1940; Bhatia, 1999; Lombroso and Fischman, 1999; Vercueil, 2000; Lee *et al.*, 2004; Engelen and Tijssen, 2005; Friedman *et al.*, 2009; Ghezzi *et al.*, 2009; van Rootselaar *et al.*, 2009; Benz *et al.*, 2012; Pons *et al.*, 2012). The gene responsible for PNKD was identified as the *MR-1* gene in 2004, but it is now referred to as *PNKD* (Raskind *et al.*, 1998; Lee *et al.*, 2004; Rainier *et al.*, 2004)*.* To date three mutations in this gene have been reported; p.A7V, p.A9V and p.A33P, the first two of which have been found in multiple unrelated patients (Lee *et al.*, 2004; Friedman *et al.*, 2009; Ghezzi *et al.*, 2009; Shen *et al.*, 2011; Pons *et al.*, 2012; Erro *et al.*, 2014). Recent work from Shen *et al.*, (2015) has shown that PNKD interacts with the synaptic active zone proteins RAB-interacting molecule (RIM)1 and RIM2, and modulates neurotransmitter release. The mutant protein is less effective at inhibiting exocytosis.

Lance (1977) described a family with exercise-induced dystonia with attacks lasting between 5 and 30 min, once or twice per month. This disorder is now termed PED (Lance, 1977). PED is thought to be the rarest of the three paroxysmal movement disorders, where attacks are induced by physical exertion after long periods of exercise. The condition can be associated with migraine, hemiplegia, ataxia and epilepsy (Zorzi *et al.*, 2003; Bhatia, 2011). Mutations in the *SLC2A1* gene, which encodes the glucose transporter type 1 protein, have recently been found to be responsible for causing PED, often called GLUT1 deficiency syndrome 2 (Wang *et al.*, 2000; Vermeer *et al.*, 2007; Suls *et al.*, 2008). *SLC2A1* mutations also cause GLUT1 deficiency syndrome 1, a phenotypically variable syndrome that often includes ataxia, microcephaly, intellectual dysfunction, dystonia, epilepsy and low fasting glucose levels detected on CSF analysis (Wang *et al.*, 2000; Vermeer *et al.*, 2007; Suls *et al.*, 2008; Schneider *et al.*, 2009; Fung *et al.*, 2011; Gokben *et al.*, 2011; Hashimoto *et al.*, 2011; Bawazir *et al.*, 2012; Agostinelli *et al.*, 2013; Muhle *et al.*, 2013; Weller *et al.*, 2015).

The majority of published reports on paroxysmal movement disorders are single families, small series or single gene studies with little known about the gene mechanisms. Here, we carry out the first large screening study of the three main paroxysmal dyskinesia genes [the total coding regions of *SLC2A1* and *PRRT2* and exons one and two (the only exons in which mutations have been previously identified) of *PNKD*] in a large referral series of 145 paroxysmal movement disorders and in a further 53 genetically undefined patients with episodic ataxia or familial hemiplegic migraine. We identify the mutation frequency and spectrum as well as genetic and phenotypic heterogeneity, describe novel mutations, and investigate the mutation mechanisms amongst the paroxysmal dyskinesias.

## Materials and methods

Patients and unaffected family members were recruited through the laboratory with consent and ethical approval (NHNN studies 06/N076 and 07/Q0512/26); they were seen either at the National Hospital in Queen Square, or referred from other centres for genetic testing with local approval. Patients were diagnosed with a paroxysmal dyskinesia or movement disorder based on recognized criteria (Bruno *et al.*, 2004, 2007; Kinali *et al.*, 2004; Bhatia, 2011) by the authors. Acquired causes were excluded using clinical investigation prior to genetic testing. Episodic ataxia and familial hemiplegic migraine cases were negative for mutations in the *KCNA1* and *CACNA1A* genes by direct sequencing of all codons. DNA was extracted from blood of affected patients and unaffected family members using standard diagnostic laboratory methods.

### Sequencing

Polymerase chain reaction (PCR) was used to amplify the three coding exons and flanking introns of the *PRRT2* gene, the 10 coding exons and flanking introns of the *SLC2A1* gene, and the first two coding exons and flanking introns of the *PNKD* gene (Supplementary Table 1). For each gene the longest transcript was used for primer design and sequencing: *PRRT2*-001: ENST00000358758; *SLC2A1*-001: ENST00000426263; *PNKD*-001: ENST00000273077. PCR amplification was performed using 10 pmol of both forward and reverse genomic primers (synthesized by Sigma-Aldrich) and FastStart™ Taq DNA polymerase (Roche). Each purified product was then sequenced using forward or reverse primers, as well as internal sequencing primers to ensure complete coverage of in the case of exon 2 of *PRRT2* with Applied Biosystems BigDye® terminator v3.3 sequencing chemistry as per the manufacturer’s instructions. The resulting reactions were resolved on an ABI3730XL genetic analyser (Applied Biosystems) and analysed with SeqScape v2.5 software (Gene codes).

In developing our genetic analysis strategy for diagnostics we also developed a custom Illumina sequencing gene panel (Illumina Inc.). This panel included the *PRRT2*, *SLC2A1* and *PNKD* genes*.* These genes had a mean coverage of 269×, 196× and 178×, respectively and 24 samples were analysed in this way. All regions of the genes were covered and no coverage gaps had to be completed by Sanger sequencing. The analysis of data consisted of mapping the raw data to the hg19 human reference assembly using Novoalign software, and PCR duplicates were removed using the Picard software. Indels were called using the GATK package and variants annotated using SAMtools. Mutations were verified in both directions. Mutation position was labelled from the transcriptional start site of the genes, according to the standard nomenclature.

### Expression methods

Regional distribution of *PRRT2*, *SLC2A1*, *PNKD*, *KCN1A*, *SNAP25* and *CACNA1A* mRNA expression in the normal human brain was determined using microarray analysis of human post-mortem brain tissue from the UK Human Brain Expression Consortium (Trabzuni *et al.*, 2011). Brain tissues originating from 134 control Caucasian individuals were collected by the Medical Research Council (MRC) Sudden Death Brain and Tissue Bank (Edinburgh, UK). The following brain regions were included in the analysis: cerebellum, frontal cortex, hippocampus, medulla, occipital cortex, putamen, substantia nigra, temporal cortex, thalamus and white matter. Total RNA was isolated from these tissues using mRNeasy 96-well kit (Qiagen) before processing with the Ambion® WT Expression Kit and Affymetrix GeneChip Whole Transcript Sense Target Labeling Assay, and hybridization to the Affymetrix Exon 1.0 ST Array. The probe set defining each gene mRNA was determined using the Affymetrix Netaffx annotation file (HuEx-1_0-st-v2 Probe set Annotations, Release 31). The combined signal of the gene probe sets were used to determine mRNA expression.

Sequencing of *PNKD* and *PRRT2* cDNA from affected patient fibroblast mRNA was carried out to assess the presence of nonsense-mediated decay and to indicate the presence of a truncated protein in mutations that affect the last exon of the gene. Fibroblasts were first taken with informed consent and mRNA was extracted using a Qiagen miRNA kit. cDNA was synthesized from the mRNA with SuperScript® II reverse transcriptase according to the manufacturer’s protocol, 1000 ng of mRNA was used as template with random oligonucleotide primers. The *PNKD* C-terminal and the *PRRT2* (across the whole gene) of the resulting cDNA product was then amplified by 35 cycles of PCR and sequenced by the above method, using primers designed to amplify only cDNA and not genomic DNA.

## Results

**Figure 1:**
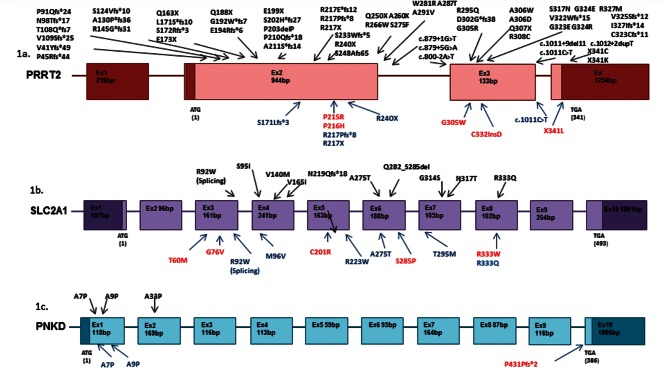
**Genetic structure and mutations in *PRRT2*, *SLC2A1* and *PNKD*.** Schematic diagrams of the *PRRT2* (**A**), *SLC2A1* (**B**) and *PNKD* (**C**) genes. In each case mutations that have been previously reported to cause a paroxysmal movement disorder are shown above the gene, and mutations found in this paper are shown below (blue have previously been reported, red are novel).

**Figure 2:**
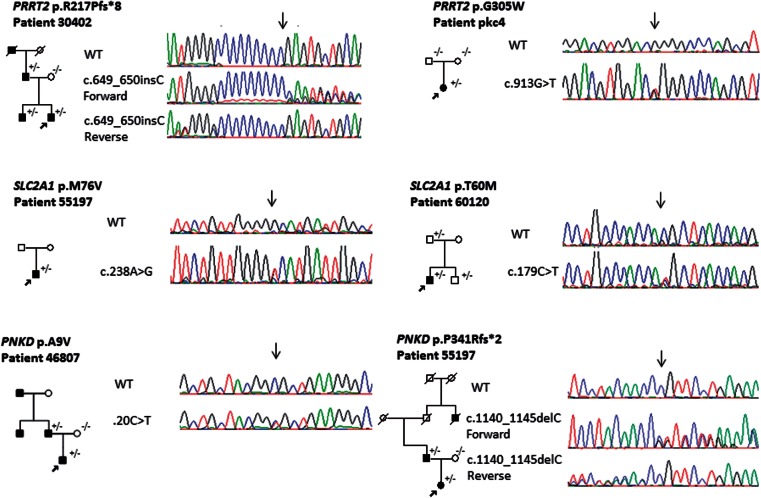
**Family tree and mutation chromatograms.** Filled symbols indicate family members that are affected, unfilled symbols are unaffected. The proband is indicated with a black arrow. +/− denotes an individual that is heterozygous for the mutation shown, −/− does not carry the mutation.

**Figure 3:**
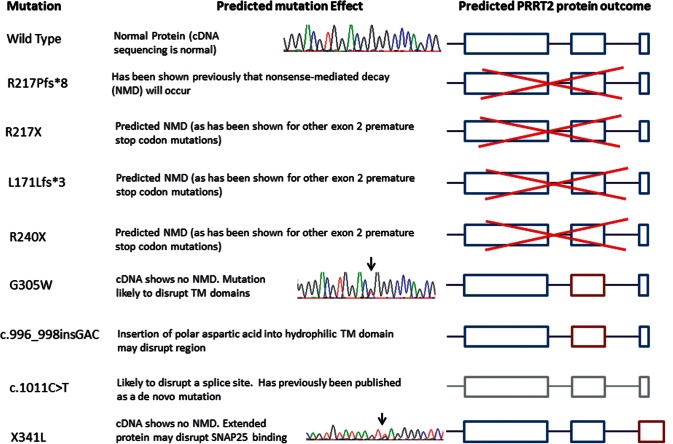
**The predicted protein consequence of mutations in the *PRRT2* gene.** Red cross = nonsense-mediated decay; burgundy outline = mutated exon; grey outline = reduced expression. Chromatograms show the presence of a mutation in mRNA, excluding the possibility of nonsense-mediated decay.

**Figure 4:**
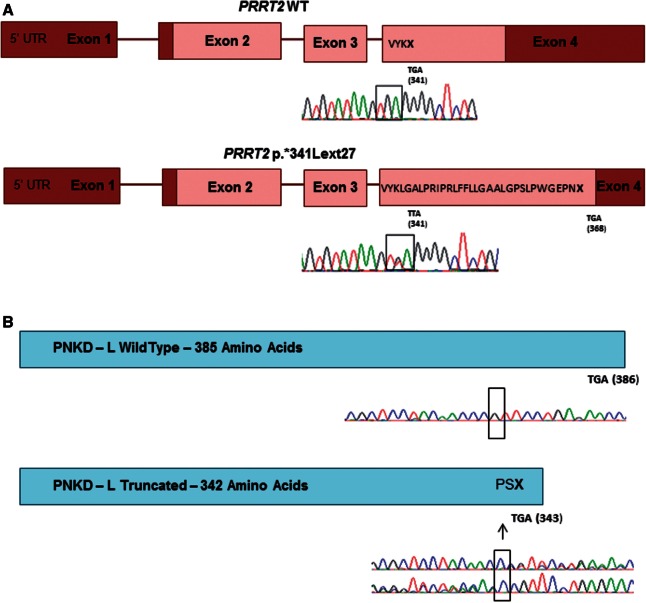
**Mutation effect in *PRRT2* and *PNKD* frameshift mutations.** (**A**) Schematic diagram of PRRT2 showing the elongation of the protein caused by p.*341Lext27, and the chromatogram identifying the mutation in the patient DNA with no NMD in mRNA from this family. (**B**) Schematic diagram of the wild-type and truncated PNKD-L, the result of the p.P341Pfs*2 mutation. The cDNA sequencing (**B**) shows the mutation was present at the mRNA level (*top* = forward sequencing, *bottom* = reverse sequencing in the *lower* figure) and so excludes the possibility of nonsense-mediated decay.

**Figure 5:**
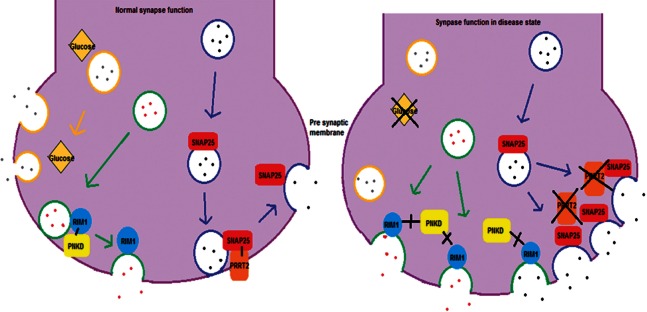
**Likely mechanism of action of paroxysmal dyskinesia genes.** A suggested mechanism for the paroxysmal dyskinesia genes, where mutations in *PRRT2*, *PNKD* and *SLC2A1* result in disruption of neurotransmitter release regulation and thus impaired synaptic release. Circles indicate presynaptic vesicles containing neurotransmitter (dots). Yellow vesicles are affected by *SLC2A1* mutations, green by *PNKD* mutations and blue by *PRRT2* mutations.

Mutations in the *PRRT2* gene were found in 53 families or sporadic cases, with nine different mutation types (Figs 1–5 and Table 1). A male to female ratio of 2:1.3 was identified in those expressing a phenotype, and the patient demographic was 56% British and a mixture of other populations accounting for the other 44%. As widely reported, by far the most common mutation (44 families, 82%) was an insertion of a cytosine into a string of nine cytosines, resulting in a frame shift mutation and premature stop codon (p.R217Pfs*8). Each of the other nine mutations accounted for one family and the majority were loss-of-function. These mutations were found in families with a number of different ethnicities and there was no common background haplotype. Four mutations were novel and two of the mutations (p.G305W and p.C332_V333insD) have only been reported by us in the past. We include them here, as well as the cases with p.R217Pfs*8 mutations, for the assessment of the frequency of *PRRT2* mutations in our cohort (Gardiner *et al.*, 2012; Silveira-Moriyama *et al.*, 2013). The p.P215R variant is also included in the mutation table; it has a frequency of <7:10 000 in the ExAC database and not seen in 488 UK control subjects. The pathogenicity of this change is still uncertain. The p.P216H variant has been found in our patient series but was also found in the UK control population at a rate of 1%. Mutations in the *PRRT2* gene were mainly associated with paroxysmal kinesigenic dyskinesia with a number of associated phenotypes (Table 1) including: (i) episodic ataxia; (ii) benign epilepsy; (iii) PED; and (iv) migraine and familial hemiplegic migraine. Fifty-one patients were part of the paroxysmal dyskinesia series and the remaining two were from the episodic ataxia and familial hemiplegic migraine series.

**Table 1:** Clinical phenotype and demographics of families and patients with *PRRT2* mutations

Patient	Ethnicity	Age at onset/current age	Affected cases and gender	Phenotype in the proband	Family history	Family members tested for segregation	Genetics	Frequency in ExAC	Previously reported (reference)
1	Somalia	12–13/24–27	1M 1F	PKD with seizures	Affected sister	Yes	p.L171Lfs*3	0	Chen *et al.*, 2011
2	British	7–8/12–16	1M 1F	PKD, one unaffected with the mutation.	Yes, affected sister, mother unaffected carrier	Yes	p.R217X	0	Liu *et al.*, 2012
3	Austrian	0.5–27/29–51	1M 1F	PKD	Yes, affected sister, father unaffected carrier	Yes	p.R217Pfs*8	0.006	Chen *et al.*, 2011; Wang *et al.*, 2011; Lee *et al.*, 2012
4	Wales/ India	6–11/18–49	4M	PKD, Migraine with aura	Yes, affected paternal grandfather, father, brother with migraine	No	p.R217Pfs*8	0.006	As above for p.R217Pfs*8
5	Ireland	8/42–45	1M 1F	PKD	Yes, affected sister	Yes	p.R217Pfs*8	0.006	
6	British	1–6/12–62	1M 1F	PKD, several individuals with HM and classical migraine	Yes, autosomal dominant family	Yes	p.R217Pfs*8	0.006	
7	British	0.5–8/12–52	2M 2F	Benign familial infantile epilepsy, HM	Yes, father, sister and cousins affected with HM	No	p.R217Pfs*8	0.006	
8	Pakistan	14/31–33	1M 1F	PKD	Yes, affected sister	No	p.R217Pfs*8	0.006	
9	British	4–10/12–56	2M 2F	PKD, meningitis and recurrent seizures as a child	Yes, brother and sister possibley affected, affected mother	Yes	p.R217Pfs*8	0.006	
10	British	6–11/20–68	2M 2F	PKD with migraine	Autosomal dominant family history. Seizures in one case.	Yes	p.R217Pfs*8	0.006	
11	British	6–16/8–38	1M 1F	PKD	Probable, mother migraine	No	p.R217Pfs*8	0.006	
12	Turkey	5/16	1M	PKD	No family history	No	p.R217Pfs*8	0.006	
13	British	12/18	1F	PKD	None	No	p.R217Pfs*8	0.006	
14	British	10/12–59	1M 2F	Episodic ataxia with familial hemiplegic migraine	Yes, affected mother and children with familial hemiplegic migraine	Yes	p.R217Pfs*8	0.006	
15	Pakistan	8/40–42	2M	PKD, both brothers have migraine with aura	Yes, affected brother	No	p.R217Pfs*8	0.006	
16	Malta	8–18/25–48	1M 1F	PKD with migraine	Yes, affected mother	Yes	p.R217Pfs*8	0.006	
17	Pakistan	8/43	2M	PKD with headaches	Yes, affected twin brother	No	p.R217Pfs*8	0.006	
18	British	27/48	1M	PKD	No	No	p.R217Pfs*8	0.006	
19	Singapore	9–12/42–47	1M 1F	PKD	Yes, daughter has childhood seizures	No	p.R217Pfs*8	0.006	
20	India	6–14/12–42	3M	PED with migraine	Yes, affected brother and father, family history of seizures	Yes	p.R217Pfs*8	0.006	
21	British	0.5–30/87	3M 3F	PKD, Migraine, HM, epilepsy. Three mutation carriers asymptomatic	Yes, dominant, large number affected	Yes	p.R217Pfs*8	0.006	
22	British	12–18/14–39	1M 1F	PKD	Yes, mother has migraine	No	p.R217Pfs*8	0.006	
23	British	14/39	1M	PKD	No	No	p.R217Pfs*8	0.006	
24	India	7–13/9–60	9M 6F	PKD with seizures in many as a child. Several mutation carriers are asymptomatic.	Yes, large autosomal dominant family history	Yes	p.R217Pfs*8	0.006	
25	India	8/12–52	2M 1F	PKD with migraine	Yes, father affected and seizures in paternal aunt	Yes	p.R217Pfs*8	0.006	
26	British	8–12/28–76	5M 8F	PKD with hemiplegic migraine and seizures	Yes, large autosomal dominant family history	Yes	p.R217Pfs*8	0.006	
27	Slovakia	6–7/9–12	2F	PKD with migraine and burning hemiplegia	Yes, sister has migraine	No	p.R217Pfs*8	0.006	
28	British	6–8/12–49	1M 2F	PKD	Yes, two affected relatives	No	p.R217Pfs*8	0.006	
29	British	9/32	1F	PKD	Yes, mother with migraine, uncle with infantile convulsion	No	p.R217Pfs*8	0.006	
30	British	9/19	1F	PKD	No	No	p.R217Pfs*8	0.006	
31	British	11/20	1F	PKD	Mother is carrier, she had single episode of torticollis but no paroxysmal movement disorder	Yes	p.R217Pfs*8	0.006	
32	Pakistani	∼10/18–69	1M	PKD	Yes, affected father with PKD	Yes	p.R217Pfs*8	0.006	
33	Irish	6–12/31–59	1M 1F	Infantile convulsions with HM	Yes, Multiple affected members with PKD, infantile convulsions and/or HM	Yes	p.R217Pfs*8	0.006	
34	Irish	0.5–5/8–40	1F 2M	ICCA later PKD and migraine	Yes, affected brother, father mutation carrier but no history of ICCA	Yes	p.R217Pfs*8	0.006	
35	British	0.5/2	1M	Infantile seizures	Yes, affected father with PKD	No	p.R217Pfs*8	0.006	
36	British	39/63	1M	PKD and episodic ataxia with dysarthria	No	No	p.R217Pfs*8	0.006	
37	British	3/8	1M	PKD	No	No	p.R217Pfs*8	0.006	
38	British	12/29	1M	PKD	No	No	p.R217Pfs*8	0.006	
39	Sri Lanka	6/16	1M	PKD	N/A	No	p.R217Pfs*8	0.006	
40	Afghanistan	8/15	1M	PKD and HM	No	No	p.R217Pfs*8	0.006	
41	Pakistan	8/27	1M	PKD	N/A	No	p.R217Pfs*8	0.006	
42	Australia	5/10	2M	PKD and hemiplegic migraine	Yes, father had hemiplegic migraine	No	p.R217Pfs*8	0.006	
43	British	7–14/22–49	1M 1F	PKD	Yes, mother	Yes	p.R217Pfs*8	0.006	
44	British	14/33	1M	PKC or PKD	No	No	p.R217Pfs*8	0.006	
45	British	7–12/9–32	2F	PKC and hemiplegic migraine	Yes mother	No	p.R217Pfs*8	0.006	
46	British	8–14/12–37	1M 1F	PKC	Yes mother	No	p.R217Pfs*8	0.006	
47	British	11/21	1M	PKD	No	No	R240X	0	Cloarec *et al.*, 2012; Lee *et al.*, 2012,
48	British	NA/23	1F	PKD	No	Yes	p.G305W	0	No
49	India	14/46	2M	PKD and migraine	Yes, father had seizures as a child	No	c.997_998insATG; p.C332_V333insD	0	No
50	British	2/15	1F	HM and benign seizures	Yes, several	No	c.1011C > T (exon 3 splice site)	0.00002	Liu *et al.*, 2012
51	British	12–18/16–35	2F 2M	PKD, migraine with aura (visual and hemisensory)	Yes, family history of migraine with aura and epilepsy	Yes	p.*341Lext27	0	No
52	British	15/32	1F	PKD, migraine with aura	No	No	p.P215R	0.0008	Gardiner *et al.*, 2012
53	Mauritius	8–24/16–62	2F 6M	PKD and migraine	Yes multiple	Yes	p.P215R	0.0008	Gardiner *et al.*, 2012

HM = hemiplegic migraine; ICCA = infantile convulsions and paroxysmal choreoathetosis. Variants were seen that cause amino acid changes E23K but this was non-pathogenic as seen in controls. P215H and R216H are of unknown pathogenicity as seen in the control population and R216H was in a patient with a definite *SLC2A1*.

Migraine and hemiplegic migraine were by far the most common associated phenotypes (Table 1). Interestingly, the majority of patients were given symptomatic treatment, mainly with carbamazepine; it has been widely reported that patients with *PRRT2*-positive PKD are more likely to respond well to the drug than patients without a mutation (Li *et al.*, 2013; Mao *et al.*, 2014). There did not appear to be a correlation between genotype and efficacy of treatment in our cohort. Initially the extended Indian families were taking phenytoin, which was then usually switched to carbamazepine, and lamotrigine in one patient. Depending on availability some of the extended Indian family patients still take phenytoin. Patient 48, who did appear to benefit from even high doses of the drugs. No treatment was being given in three families, at patients’ request. A family with episodic ataxia and one with familial hemiplegic migraine alone were identified with *PRRT2* mutations. The familial hemiplegic migraine family proband presented as an infant with infrequent seizures until age 2 years and then developed typical hemiplegic migraine attacks. The sister, father and two cousins also had classical hemiplegic migraine and the attacks in the proband persisted until now (aged 18 years) but responded to carbamazepine.

Fourteen *SLC2A1* mutations were identified in the paroxysmal dyskinesia series (10%) and one in the episodic ataxia and familial hemiplegic migraine series (Figs 1, 2 and Table 2). In general these were complex cases that had been heavily investigated prior to obtaining a genetic diagnosis. Eight had PED, often associated with other features such as epilepsy and migraine. Three had PKD (one with epilepsy) and one had PNKD, two with episodic ataxia and one with myotonia and dystonia, as discussed below. Eleven of the mutations had previously been reported as being pathogenic. The p.C201R mutation has not before been identified but presented with a PNKD phenotype and was present in the affected mother. p.C201R is not present in population databases, but is not well conserved and predicted to be benign by PolyPhen-2 but damaging by SIFT. p.T60M is present in 0.00015% of the population, is moderately conserved and is predicted to be damaging by PolyPhen-2 but tolerated by SIFT. This mutation has been reported in association with seizures in the past but like in our family, there was reduced penetrance. Patient 66 had sequence variants in both *SLC2A1* and *PRRT2* (p.R333Q and p.P216H, respectively) and a PKD phenotype, but the *PRRT2* mutation is unlikely to be pathogenic as it is present in 1% of controls we analysed, and the p.R333Q mutation has been reported previously as pathogenic.

**Table 2:** Clinical phenotype and demographics of families and patients with *SLC2A1* mutations

Patient	Ethnicity	Age at onset/current age	Affected cases and gender	Phenotypic description	Family history	Family members tested for segregation	CSF glucose: blood ratio	Genetics	Frequency in ExAC	Previously reported (reference)
54	British	5/40	1F	Exercise induced dystonia, seizures and hemiplegic migraine	No	No	Low, 0.5	p.G18R	0	Weller *et al.*, 2015
55	Asian	1/9	1M	Frequent paroxysmal episodes of unsteadiness, headaches, nystagmus, vomiting. MRI normal. Present in unaffected father and brother	No	Yes	Normal	p.T60M	0.00002	Arsov *et al.*, 2012
56	British	8/28	1M	Myotonia and dystonia	No	No	Normal	p.G76V	0	No
57	British	2/25	1F	PED	No	No	N/D	p.R91W	0	Schneider *et al.*, 2009
58	British	6-13/18-78	2M 2F	PKD in three cases, PED in one. Attacks typical of PKD	Yes, family history of migraine.	No	Normal	p.R92W	0	No
59	British	11/46	3F	Severe PED and PKD	Yes, AD family history	No	Low, 0.4	p.M96V	0	Leen *et al.*, 2010
60	British	Teens/49	1M 2F	PNKD	Affected mother	Yes	Normal	p.C201R	0	No
61	British	8/24	1M	PKD with epilepsy	No	No	N/A	p.R223W	0	Leen *et al.*, 2010
62	British	12/42	1M 1F	PED	Dominant inheritance	Yes	Normal	p.A275T	0	Weber *et al.*, 2008
63	British	15/28	1F	PED and seizures	No	No	Low 0.55	p.S285P	0	No
64	Ireland	4/17	1M 2F	EA2, early absence seizures	No	No	N/A	p.T295M	0	Weber *et al.*, 2008
65	British	Child/36	1M 2F	PED	No	No	N/A	T295M	0	Weber *et al.*, 2008
66	British	5/13	1F	PKD, long and frequent episodes of dystonia and unusual tongue dystonia.	No	No	N/A	p.R333Q + PRRT2 (p.R216H)	0	Schneider *et al.*, 2009
67	British	4/54	1M 1F	PED, migraines and seizures	No	No	Low, 0.5	p.R333Q	0	Schneider *et al.*, 2009
68	British	12/26	1M 1F	PED, seizures	Daughter affected	Yes	Low, 0.5	p.R333W	0	Wang *et al.*, 2000

AD = Alzheimer’s disease; EA = episodic ataxia; HM = hemiplegic migraine; N/D = not determined.

The majority of *PRRT2* mutations are predicted to be loss-of-function and likely lead to haploinsufficiency. It has been previously demonstrated that mutations p.Q163X, p.G192WfsX8 and p.R217PfsX8 result in nonsense-mediated decay (Wu *et al.*, 2014). This is not the case for all mutations as cDNA created from two of our mutations; p.G305W and p.*341Lext27 (a stop codon mutation extending the protein, HGVS standard nomenclature used; den Dunnen and Antonarakis, 2000) do not affect mRNA or lead to a longer transcript (Figs 3 and 4A). The mechanism behind these two mutations is likely to be the same as those causing nonsense-mediated decay with lack of association in the SNAP25/SNARE complex and greater vesicle release (Fig. 5). *SLC2A1* mutations were associated with a wide spectrum of clinical features. Family 56 was identified with a novel heterozygous mutation at p.G76V that was not present in 488 controls and 6502 exomes in the exome variant server. This patient was a 26-year-old, diagnosed with attention-deficit hyperactivity disorder as a child and since then has had episodes of ‘wobbly’ eyes, legs and arms, and abnormal arm posturing that last 5–10 min, several times per day. Triggers for these episodes included tiredness, sudden movement, intercurrent infection or illness and excitement. He experienced episodes of weakness and painful cramps in his hands and his legs. He has tried carbamazepine, which helped a little, and acetazolamide may have helped reduce the frequency of these attacks. He underwent repeat long exercise testing (McManis) and this showed significant decrement, accompanied by weakness of the exercised hand muscles. This was most unexpected given that SLC2A1 is best known as a brain transporter; however, there is some evidence of the protein having an additional important role in skeletal muscle (Andrisse *et al.*, 2014). This result was repeated and abnormal spanning over several years. The significant decrement on McManis testing ranged from 51–66%. The clinical diagnosis at that time suggested a periodic paralysis phenotype but the movement disorder was not consistent with this.

In the *PNKD* gene, four mutations were identified (Figs 1–3 and Table 3). Three were in the paroxysmal dyskinesia series and one in a familial hemiplegic migraine family. The mutations associated with paroxysmal dyskinesias were in phenotypically typical PNKD families with non-kinesigenic precipitants such as stress or strong coffee. These mutations have been reported in the past and these were in two unrelated families with p.A7V and one with p.A9V. In the familial hemiplegic migraine family the mutation was novel and the female proband presented at 42 years of age with a typical attack of hemiplegic migraine with headache, abnormal vision and left-sided motor and sensory weakness that lasted for 45 min to an hour in duration. She had a normal MRI shortly after the event and other cardiac investigations were unremarkable, and the hemiplegic migraine resolved. A few months later she had a similar hemiplegic migraine attack. Her paternal great uncle and father had similar attacks. Her father presented at a similar age and to date has had over 50 hemiplegic migraine attacks, often without a headache. He has presented to the emergency department many times concerned that this was a stroke and has been extensively worked up but imaging and other investigations have been normal. A heterozygous mutation of c.1022delC; p.P341fs*2 was identified in the *PNKD* gene in the proband and father, not in the mother. We analysed cDNA, from mRNA extracted from patient fibroblasts. The deletion was present in the mRNA, indicating that nonsense-mediated decay would not occur, although nonsense-mediated decay is dependent on cell type and therefore it is possible that it could occur in neurons. This mutation therefore caused the formation of a truncated *PNKD* in the mRNA (Figs 3 and 4B). Although functional work was not carried out the truncating effect of this mutation is likely to have an abnormal effect on exocytosis due to impaired interaction between PNKD and RIM/RIM1 (Fig. 5).

**Table 3:** Clinical phenotypes of the four *PNKD* probands and mutations

*n*	Ethnicity	Age at onset/current age	Sex	Phenotypic description	Family history	Family members tested for segregation	Genetics	Frequency in ExAC	Previously reported (reference)
69	German	Teens/20s	3F 3M	PKD	Four generation large family	Yes	p.A7V	0	Lee *et al.*, 2004; Rainier *et al.*, 2004
70	British	16/32	2M 2F	PNKD with atypical features	Yes, father, paternal uncle and grandmother	Yes	p.A9V	0	Lee *et al.*, 2004; Rainier *et al.*, 2004
71	British	8-22/20-64	17M 10F	PNKD	Several affected over three generations	Yes	p.A9V	0	Lee *et al.*, 2004; Rainier *et al.*, 2004
72	British	30-34/44-78	2M 1F	Familial hemiplegic migraine	Father, great-uncle and proband over three generations	Yes	c.1022delC p.Pro341fs	0	No

## Discussion

High prevalences of *PRRT2*, *SLC2A1* and *PNKD* mutations were identified in this large, mainly London based paroxysmal movement disorder referral series. Although we have a multi-ethnic population the results corroborate smaller individual gene series (Fig. 1 and Tables 1–3). There was a spectrum of clinical features and many patients had additional clinical features such as seizures. The frequency of migraine and hemiplegic migraine was highly associated with these phenotypes although this is also common in the general population. Some individuals in the extended PKD families did not have a movement disorder at all or were affected by seizures or hemiplegic migraine alone. The usual mechanism for *PRRT2* mutations is loss of function due to nonsense-mediated decay, leading to haploinsufficiency (Figs 2–5) and likely lead to a lack of SNAP25/SNARE interaction and increased vesicle release. Segregating *PRRT2* missense mutations were also identified where there was no change in the *PRRT2* mRNA, but we expect a loss of SNAP25/SNARE interaction or prevention of the PRRT2 protein from anchoring to the presynaptic membrane, and thus leading to a similar lack of inhibition of vesicle release due to reduced tethering (Fig. 5).

Fewer mutations were identified in the *SLC2A1* and *PNKD* genes, and primarily in patients with PKD and PNKD (Fig. 1 and Table 2). The patients with *SLC2A1* mutations had the broadest spectrum of clinical phenotypes. There was overlap clinically with PKD (as in the p.R223W family) and PNKD (as with the p.C210R family). This group were the most extensively investigated before a genetic diagnosis was sought, and fasting CSF glucose was frequently low in affected individuals with a more complex phenotype associated with seizures but usually normal with a movement disorder alone. There was also a greater rate of an incorrect clinical diagnosis and overlap with other channelopathies, as with the family with the p.G76V mutation and abnormal McManis testing, and in the family with the p.R333Q mutation and unusual tongue dystonia as part of the phenotype. These families are similar to those first described in 1892 as atypical Thomsen’s disease (Kure, 1892). The p.R333Q had an additional variant in the *PRRT2* gene (p.P216H), which may be benign or modifying the effect of the p.R333Q mutation. In addition there was evidence of reduced penetrance in *SLC2A1*, most clearly in the family with the p.T60M mutation that presented with paroxysmal attacks, headaches and nystagmus where the father and brother had the mutation but were unaffected (see family tree, Fig. 2). The p.T60M mutation has previously been identified in idiopathic epilepsy, further extending the heterogeneity.

In the episodic ataxia cohort, one family was identified with a mutation in the *PRRT2* gene, one with a defect in the *SLC2A1* gene and two familial hemiplegic migraine families were identified, one with a *PRRT2* mutation and one with a novel *PNKD* mutation. The familial hemiplegic migraine families were of most interest as they have a typical phenotype and the mutations segregate in the family. The novel *PNKD* mutation is a frameshift deletion located in exon 10, which is predicted to cause a truncated protein, this segregated with the disease, predicted pathogenic and was not identified in controls (Figs 2, 3 and 4B). Alternate splicing of the *PNKD* gene results in three isoforms of the protein of varying length; PNKD-S, PNKD-M (both expressed ubiquitously), and PNKD-L (expressed in the CNS) (Shen *et al.*, 2011). All previously reported mutations are located in the 5’ end of the gene, found in both PNKD-L and PNKD-S but not PNKD-M. This mutation, instead affects PNKD-L and PNKD-M and the location and truncating effect of the change in shortening the PNKD protein is likely to lead to reduced RIM/RIM1 binding (Shen *et al.*, 2015) in the SNARE complex and abnormal vesicle release (Fig. 5).

While there is a great deal more to be understood, it seems likely that these three paroxysmal genes are acting on the presynaptic terminal, possibly with overlapping pathways, and thus result in a similar dysregulated and possibly increased vesicular release. Although there is clinical overlap, there are also additional clinical features. This overlap is seen in the brain expression patterns where genes with a similar mechanism have identical regional expression patterns (Supplementary Fig. 1) as for *PRRT2*, *SNAP25*, *KCNA1* and *CACNA1A* (all presynaptic) where they share highest expression levels in the cerebellum, and frontal, temporal and occipital cortices as compared with *SLC2A1* and *PNKD*. This could explain the subtle phenotypic differences and the regional effect on vesicle release. It has recently been reported that overexpression of wild-type *PNKD* in rat hippocampal cultures reduced neurotransmitter release in comparison to an empty vector, whereas overexpression of mutant *PNKD* did not. This suggested that PNKD also has a role in regulating presynaptic exocytosis (Lee *et al.*, 2015). It is also known that PRRT2 interacts with SNAP25, a protein important in facilitating synaptic exocytosis (Lee *et al.*, 2015). Therefore, we suggest a possible disease mechanism whereby both PNKD and PRRT2 perform similar roles in restricting synaptic exocytosis. Disease-causing mutations that either reduce levels of PRRT2 or disrupt PNKD function reduce this restriction and result in excessive neurotransmitter release (Fig. 5). It is unclear how *SLC2A1* mutations contribute to this theory, but it has been shown that they result in reduced glucose transport into the brain, so perhaps glucose is also involved in the regulation of exocytosis. The functional consequence of the regional expression patterns remains to be seen but may indicate that SLC2A1 and PNKD pathways are more closely related to dystonic genes located in the basal ganglia and brainstem.

Little is known about how disruption of these proteins results in migraine, a clinical manifestation that has been seen frequently here and elsewhere. However, in a recent study, transgenic mice with human monogenic migraine gene mutations (thus mimicking the types of migraine seen in this cohort) were shown to display increased glutamatergic neurotransmission and cerebral hyperexcitability (Ferrari *et al.*, 2015). This finding indicates that the lack of neurotransmitter release regulation postulated here could also result in the migraine exhibited. There is clearly a large pathophysiological overlap between all of these related neurological disorders, which required further investigation to be understood more fully.

Overall this work reveals a wide spectrum of mutations and phenotypes and has expanded the broad phenotypic spectrum of these paroxysmal movement disorders, suggesting where possible, as part of the investigative work-up, all three genes should be analysed in these conditions. We also highlight novel mutations and a likely distinct mechanism for 3’ *PNKD* mutations that lead to PNKD-L dysregulation. There is genetic and phenotypic overlap amongst other episodic movement disorders with episodic ataxia, the neuronal channelopathies and familial hemiplegic migraine all being identified with defects in these three genes.

## Supplementary Material

Supplementary Table 1

## Abbreviations

PED: paroxysmal exercise-induced dyskinesia
PKD: paroxysmal kinesigenic dyskinesia
PNKD: paroxysmal non-kinesigenic dyskinesia
