# Supplementary material for: The clinical and genetic heterogeneity of paroxysmal dyskinesias
Source: Brain. 2015 Nov 18;138(12):3567–80. doi: 10.1093/brain/awv310 (PMC4655345; doi:10.1093/brain/awv310)

Supplementary Figure 6 Immuno-Electron microscopy of vascular amyloid displacing astrocyte endfeet

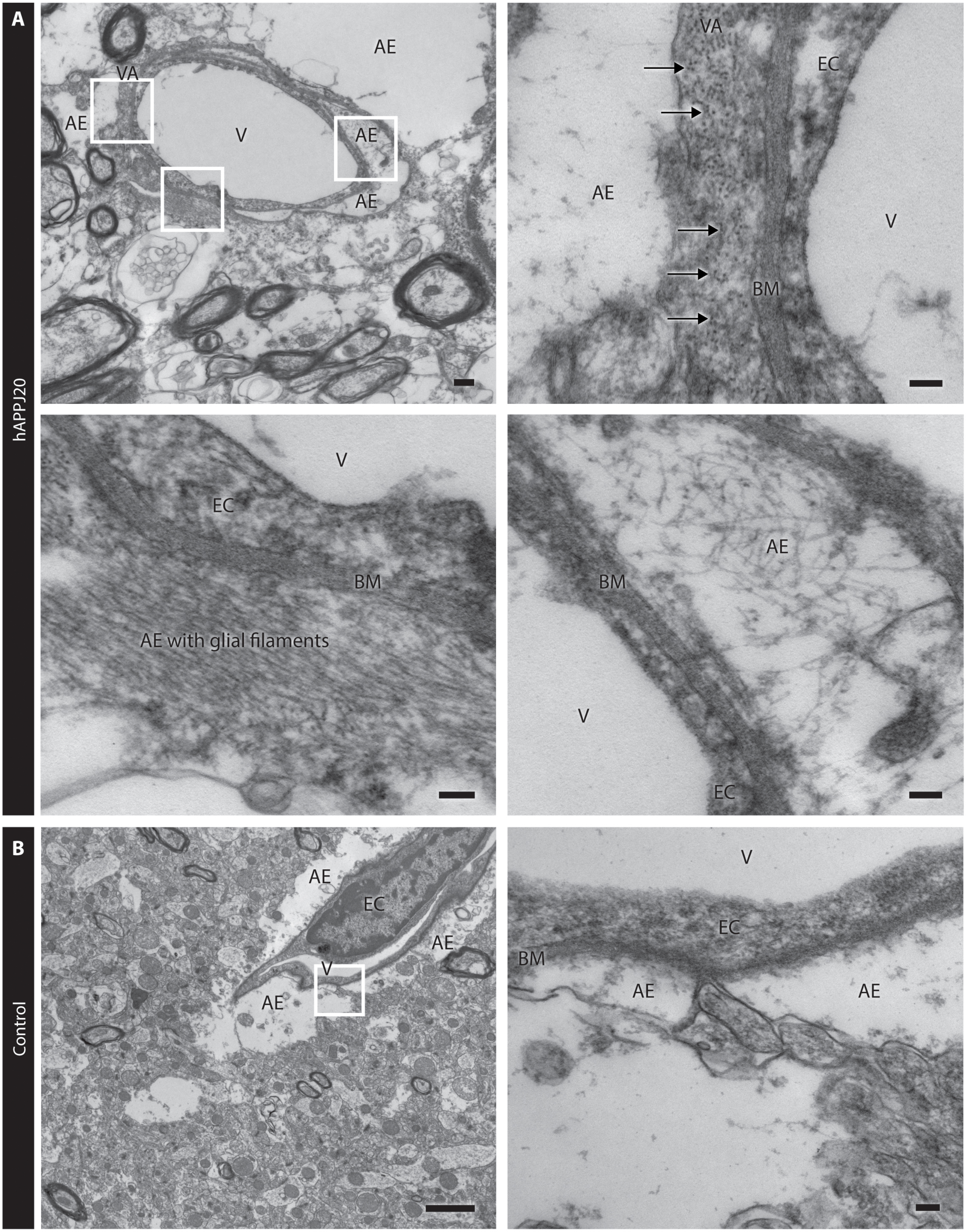

Supplement: Supplementary Table 1 [file suppl_data.zip › brain-2015-00380-File013.pdf]
