# Supplementary material for: The clinical and genetic heterogeneity of paroxysmal dyskinesias
Source: Brain. 2015 Nov 18;138(12):3567–80. doi: 10.1093/brain/awv310 (PMC4655345; doi:10.1093/brain/awv310)
Supplement: Supplementary Table 1 [file suppl_data.zip › brain-2015-00380-File016.pdf]

Supplemental Table 1

animal number	age at time of experiment	genotype	sex	experiment
#645	14 months of age	hAPPJ20	M	slices t-ACPD, NE (Fig. 3), slices PGE2 (Suppl. Fig. 8C)
#659		hAPPJ20	F	
#662		hAPPJ20	F	
#670		hAPPJ20	F	
#530		hAPPJ20	M	
#528		hAPPJ20	M	
#646		control	M	
#660		control	F	
#661		control	F	
#524		control	M	
#529		control	M	
#522		control	M	
#523		control	M	

#911	15 months of age	hAPPJ20	F	2P imaging (Fig. 1, Suppl. Fig. 2), slices ET-1 and U46619 (Fig. 5, Suppl. Fig. 8A-C)
#671		hAPPJ20	F	
#679		hAPPJ20	F	
#681		hAPPJ20	F	
#694		hAPPJ20	F	
#680		control	F	
#682		control	F	
#695		control	F	
#672		control	F	

#699	19 months of age	hAPPJ20	F	2P imaging (Fig. 1, Suppl. Fig. 2), EM (data not shown)
#700		hAPPJ20	F	
#701		hAPPJ20	F	
#708		hAPPJ20	F	
#715		hAPPJ20	F	
#697		control	F	
#698		control	F	
#709		control	F	
#710		control	F	
#716		control	F	

#910	27-29 months of age	hAPPJ20	M	EM (Fig. 2I-K, Suppl. Fig. 4)
#917		hAPPJ20	M	
#919		hAPPJ20	M	
#908		control	F	
#913		control	F	
#916		control	M	

#943	27 months of age	hAPPJ20	M	Confocal images (Fig. 2B-D), Astrocyte Ca uncaging (Fig. 4, Suppl. Fig. 1)
#963		hAPPJ20	F	
#721		hAPPJ20	M	
#901		hAPPJ20	M	
#909		hAPPJ20	M	
#914		hAPPJ20	F	
#931		hAPPJ20	M	
#937		control	M	
#959		control	F	
#722		control	M	
#925		control	F	

#1046	30 months of age	hAPPJ20	M	Fig. 6
#1057		hAPPJ20	M	
#972		hAPPJ20	F	

#3968	3 months of age	hAPPJ20	F	IHC (Fig. 2E-H)
#3970		hAPPJ20	M	
#3972		hAPPJ20	F	
#3969		control	F	
#3971		control	F	
#3973		control	M	

#351	9 months of age	hAPPJ20	M	IHC (Fig. 2E-H)
#353		hAPPJ20	M	
#358		hAPPJ20	F	
#350		control	M	
#352		control	M	
#357		control	F	

#306	12 months of age	hAPPJ20	F	IHC (Fig. 2E-H)
#308		hAPPJ20	F	
#305		control	F	
#311		control	F	

#323	16 months of age	hAPPJ20	M	IHC (Fig. 2E-H)
#318		hAPPJ20	M	
#322		hAPPJ20	M	
#321		control	M	
#326		control	M	
#340		control	F	

#973	29 months of age	hAPPJ20	F	IHC (Fig. 2E-H, Suppl. Fig. 3,6,7), Immuno-EM (Suppl. Fig. 5),
#977		hAPPJ20	F	
#978		hAPPJ20	F	
#975		control	F	
#983		control	M	
#985		control	M	
