# Supplementary material for: The clinical and genetic heterogeneity of paroxysmal dyskinesias
Source: Brain. 2015 Nov 18;138(12):3567–80. doi: 10.1093/brain/awv310 (PMC4655345; doi:10.1093/brain/awv310)
Supplement: Supplementary Table 1 [file suppl_data.zip › brain-2015-00380-File028.pdf]

Movie 1 Automated measurements of changes in vessel diameter using motion tracking system. Vessels in *ex vivo* slices were labeled with Alexa Fluor 633 Hydrazide (cyan) to label arteriole walls, Rhod-2 AM (red) which is taken up by astrocytes, and DMNPE-4 caged Ca^{2+}. Astrocytes were stimulated using a laser pulse to uncage Ca^{2+} and changes in vessel diameter in response to Ca^{2+} uncaging were calculated by automatically tracking arteriole wall movement (shown by the white lines) using After Effects software (Adobe Systems, Inc.). Distance between tracking points (in pixels) was converted to µm and then displayed as an overlay on corresponding frames.

Movie 2 3D reconstruction of the cerebral vasculature and associated vascular amyloid. Multi-photon optical slice data was captured through a chronic cranial window of a live 15-month-old hAPPJ20 mouse where TRITC-Dextran (red) labeled the lumen of the cerebrovasculature and methoxy-XO4 (green) labeled the vascular amyloid. 3D reconstructions made using Imaris software show vascular amyloid wrapping around vessels in complete or incomplete ring-like structures.

Movie 3 Calcium uncaging in astrocytes induced vasoconstriction in acute slices of control mice. Acute slices from 27-29-month-old control animals were loaded with the Ca^{2+} indicator Rhod-2 AM (red) and the Ca^{2+} cage DMNPE-4. Arterioles were visualized with the dye Alexa Fluor 633 Hydrazide (cyan), which labels the elastin layer of arterial vessels $\geq$10 µm in diameter. Animals were injected with methoxy-XO4 i.p.12 h prior to the experiment in order to label vascular amyloid (green). We never observed vascular amyloid or amyloid plaques in control animals. Uncaging in astrocytes or astrocyte endfeet (indicated by a white dot) was immediately followed by constriction of the vessel.

Movie 4 Calcium uncaging in astrocytes induced vasoconstriction of amyloid-free vessels in acute slices of hAPPJ20 mice. Acute slices from 27-29-month-old hAPPJ20 animals were loaded with the Ca^{2+} indicator Rhod-2 AM (red) and the Ca^{2+} cage DMNPE-4. Arterioles were visualized with the dye Alexa Fluor 633 Hydrazide (cyan), which labels the elastin layer of arterial vessels of ≥10 µm in diameter. Animals were injected with methoxy-XO4 i.p.12 h prior to the experiment in order to label vascular amyloid (green). Vessels or vessel segments free of vascular amyloid were chosen. Uncaging (indicated by a white dot) was targeted to astrocyte endfeet at an amyloid-free portion of the vessel. The vessel constricted strongly in response to the stimulus.

Movie 5 Calcium uncaging in astrocytes of amyloid-bearing vessels in acute slices of hAPPJ20 mice is impaired. Acute slices from 27-29-month-old hAPPJ20 animals were loaded with the Ca^{2+} indicator Rhod-2 AM (red) and the Ca^{2+} cage DMNPE-4. Arterioles were visualized with the dye Alexa Fluor 633 Hydrazide (cyan), which labels the elastin layer of arterial vessels of ≥10 µm in diameter. Animals were injected with methoxy-XO4 i.p.12 h prior to the experiment in order to label vascular amyloid (green). Vessels or vessel areas covered by vascular amyloid were chosen. Uncaging (indicated by a white dot) was targeted to astrocyte endfeet surrounding the vascular amyloid. While the vessel constricted slightly, this response was reduced when compared to control vessels or hAPPJ20 vessels free of vascular amyloid.

Movie 6 *In vivo* laser-stimulation of vascular smooth muscle cells induced constriction of amyloid-free vessels in hAPPJ20 mice. hAPPJ20 mice at 30 months of age were imaged through a cranial window. Animals were injected with Methoxy-XO4 i.p.12 h prior to the experiment in order to label vascular amyloid (green). The cerebral vasculature

was labeled by retro-orbital injection of TRITC-Dextran immediately before the imaging session. Arterioles of ≥10 µm in diameter were visualized by retro-orbital injections of the dye Alexa Fluor 633 Hydrazide (cyan). Vessels or vessel areas free of vascular amyloid were chosen. The VSMCs containing media layer of the arterial wall was targeted for laser-stimulation by measuring 4 µm from the luminal surface. In response to this stimulation (indicated by a white dot) the vessel rapidly constricts.

Movie 7 Impaired vascular response to *in vivo* laser-stimulation of vascular smooth muscle cells around amyloid-bearing vessels in hAPPJ20 mice. hAPPJ20 mice at 30 months of age were imaged through a cranial window. Animals were injected with Methoxy-XO4 i.p.12 h prior to the experiment in order to label vascular amyloid (green). The cerebral vasculature was labeled by retro-orbital injection of TRITC-Dextran immediately before the imaging session. Arterioles of ≥10 µm in diameter were visualized by retro-orbital injections of the dye Alexa Fluor 633 Hydrazide (cyan). Vessels or vessel areas covered by vascular amyloid were chosen. The VSMCs containing media layer of the arterial wall was targeted for laser-stimulation by measuring 4 µm from the luminal surface. Vessel response to this stimulus (indicated by a white dot) in areas covered by vascular amyloid was diminished.

Movie 8 *In vivo* laser-stimulation of vascular smooth muscle cells reveals exoskeleton effect of vascular amyloid in hAPPJ20 mice. hAPPJ20 mice at 30 months of age were imaged through a cranial window. Animals were injected with Methoxy-XO4 i.p.12 h prior to the experiment in order to label vascular amyloid (green). The cerebral vasculature was labeled by retro-orbital injection of TRITC-Dextran immediately before the imaging session. Arterioles of ≥10 µm in diameter were visualized by retro-orbital injections

of the dye Alexa Fluor 633 Hydrazide (cyan). This vessel is covered by vascular amyloid, which is interrupted by gaps in some places. VSMC stimulation (indicated by a white dot) induces stronger constrictions in these amyloid-free areas, while the areas with vascular amyloid respond less.

Supplementary Figure 1 Calcium and vessel response in acute slices over time. To assess the vessel response during confocal imaging, vessel diameters and $[Ca^{2+}]_i$ increases were measured in acute slices from 27-29-month-old animals after uncaging (red) and without uncaging (blue). While a slow, minor constriction was observed in both control (A) and hAPPJ20 (B) vessels even without uncaging, no increase in $[Ca^{2+}]_i$ was observed in astrocytes. It is possible that this approximately 10% change in vessel diameter resulted from laser stimulation of vascular smooth muscle cells over time. Note that maximal constrictions typically recorded after uncaging were much larger.

Supplementary Figure 2 Volumetric 3D reconstructions of the cerebral vasculature and vascular amyloid in hAPPJ20 and control mice. Volumetric 3D reconstructions were created using the Imaris 7.5.2 software (Bitplane Scientific Software) from *in vivo* two-photon optical sections captured through a chronic cranial window of 15–19-month-old animals. Images were taken at a pixel resolution of 800x800 with an optical section thickness of 0.5µm. Separate objects were created based on methoxy-XO4 (Aβ) and TRITC-Dextran (vasculature) fluorescence and then quantified volumetrically.

Supplementary Figure 3 Aquaporin-4 is downregulated in astrocyte endfeet of hAPPJ20 mice. (**A,B**) Astrocytes were labeled with GFAP (green) and GFAP-positive endfeet were found surrounding larger CD31+ blood vessels in both hAPPJ20 and control mice, age 29 months. Aquaporin-4 (AQ4, red) was normally expressed exclusively in astrocyte endfeet surrounding blood vessels, with overlapping expression of GFAP and AQ4 in endfeet around larger cortical vessels (**B**), but this localized expression pattern was lost in hAPPJ20 mice (**A**). Images were acquired using a confocal microscope on identical settings. Displays show a single 0.4 μm z-plane. Scale 20 μm. (**C,D**) Confocal images were digitally overexposed to assess the relationship between AQ4-labeled endfeet (red) and vascular amyloid labeled by Aβ immunostaining (green) around the CD31+ vasculature (white) in 29-month-old mice. Vessel areas covered by vascular amyloid in hAPPJ20 mice lacked AQ4-labeled endfeet (**C**) while AQ4+ endfeet surrounded vessels in control animals (**D**). Displays show a single 0.4 μm z-plane. Scale 10 μm. (**E**) Ratio between endfoot labeling and cytoplasmic AQ4 expression in control and hAPPJ20 29-month-old animals (control, 4.061 ± 0.41, n = 38 vessels from 3 animals; hAPPJ20, 2.982 ± 0.4, n = 41 vessels from 3 animals; P = 0.0042, two-tailed, Mann-Whitney test). (**F**) Intensity of cytoplasmic AQ4 immunohistochemistry in control and hAPPJ20 29-month-old animals (control, 30.75 ± 2.54 intensity units, n = 38 vessels from 3 animals; hAPPJ20, 46.19 ± 4.49 intensity units, n = 41 vessels from 3 animals; P = 0.046, two-tailed, Mann-Whitney test).

Supplementary Figure 4 Astrocyte endfeet are displaced by vascular amyloid at some vessels in hAPPJ20 mice. Electron micrographs in hAPPJ20 mice were compared to age-matched controls at 19 and 29 months of age. Images presented here are from 29-month-old

animals. **(A)** Vascular amyloid (vA) in direct contact with the vessel (V) wall (see Figure 2I–K for color-coded versions of these panels) while astrocyte endfeet (AE) are found on top of the vascular amyloid. Vascular amyloid is noticeably darker (i.e., more electron dense) than structures normally found surrounding vessels (see also (Yang *et al.*, 2011)). Astrocyte endfeet typically have very low electron density and contain filaments and glycogen granules. High resolution imaging of the vessel shows remnants of astrocyte endfeet on the vessel (arrows). The basement membrane, a normally highly organized ~ 50 nm structure surrounding vessels (**B-F**) appears irregular. No membrane can be made out on top of the basement membrane between the two processes labeled with arrows. Instead diffuse amyloid contacts the vessel directly. Note the tight junction connecting two endothelial cells in the center of the image (red arrow head). Scale of zoom in panel 250 nm. (**B)** In this example, astrocyte endfeet are in contact with parts of the vessel while other parts are covered with vascular amyloid. The border between astrocyte endfoot and amyloid is more defined than in example **A**. The border of the basement membrane seems to be more diffuse and less organized than in the neighboring area covered by the endfoot. Scale of zoom in panel 250 nm. (**C,D)** Lipid granules were found dispersed throughout the tissue and in close proximity or direct contact with vessel walls of hAPPJ20 and control mice at two years of age suggesting that this is an age-related phenomenon rather than related to the mutations in hAPPJ20 mice. (**E,F)** The majority of vessels in control mice are surrounded and fully covered by astrocyte endfeet.

Supplementary Figure 5 Astrocyte densities were unchanged in hAPPJ20 mice. Immunohistochemistry for S100 (red) and GFAP (green) in 29-month-old control (**A**) and hAPPJ20 (**B**) mice. (**C**) Quantification of astrocyte densities based on S100 labeling.

Astrocyte cell bodies were counted in confocal images of 5 different brain slices of each animal, with n = 3 animals of each genotype; P = 0.9824, two-tailed student t-test. Scale, 100 μm.

Supplementary Figure 6 Immuno-Electron microscopy of vascular amyloid displacing endfeet. (**A**) Electron micrographs showing examples of Aβ-immunoreactive elements (arrows) unambiguously identifying vascular amyloid on a vessel (V) of a 29 months old hAPPJ20 brain. A part of the vessel is covered by vascular amyloid which displaced the astrocyte endfoot (AE) while other vessel areas are in direct contact with astrocyte endfeet. Some endfeet contain a large amount of glial filaments. Vascular amyloid is in direct contact with the basement membrane (BM) surrounding the vessel. (**B**) Electron micrographs of a vessel in an age-matched control brain presenting with a lack of labeling and astrocyte endfeet in direct contact with endothelial cells (EC).

Supplementary Figure 7 Vascular amyloid displaces astrocyte endfeet from the vascular wall in hAPPJ20 mice. (**A**) Vascular amyloid (green) deposits on vessel walls below $GFAP^+$ astrocyte endfeet in 29-month-old hAPPJ20 mice. Upper panels represent a single optical plane of 0.4 μm, lower panel shows a 30 μm z projection. (**B**) Vessel covered by $GFAP^+$ endfeet in age-matched control animal. Scale, 20 μm. (**C,D**) Labeling of the vasculature (red) in 3-month-old hAPPJ20 and control animals shows that only a subset of larger vessels are covered by $GFAP^+$ astrocyte endfeet (green) while the label is not detected in astrocyte endfeet (detected by labeling with AQ4, data not shown) on most cerebral vessels in the cortical gray matter. Scale, 100 μm.

Supplementary Figure 8 Baseline vessel diameters were unchanged in hAPPJ20 mice. Baseline vessel diameters were compared in 27–29-month-old animals after stratifying vessels below 7 µm in diameter as capillaries and vessels above 7 µm in diameter as arterioles. No significant change was observed between capillaries (control, 4.09 ± 0.19 µm, n = 50 vs. hAPPJ20, 4.73 ± 0.26 µm, n = 31; Two-way ANOVA with Holm-Sidak's multiple comparisons test, P = 0.8884), or between arterioles (control, 14.38 ± 1.04 µm, n = 50 vs. hAPPJ20, 15.77 ± 1.18 µm, n = 61; Two-way ANOVA with Holm-
