# Supplementary figures and images for: The clinical and genetic heterogeneity of paroxysmal dyskinesias
Source: Brain. 2015 Nov 18;138(12):3567–80. doi: 10.1093/brain/awv310 (PMC4655345; doi:10.1093/brain/awv310)

Supplementary Figure 5 Astrocyte densities were unchanged in hAPPJ20 mice.

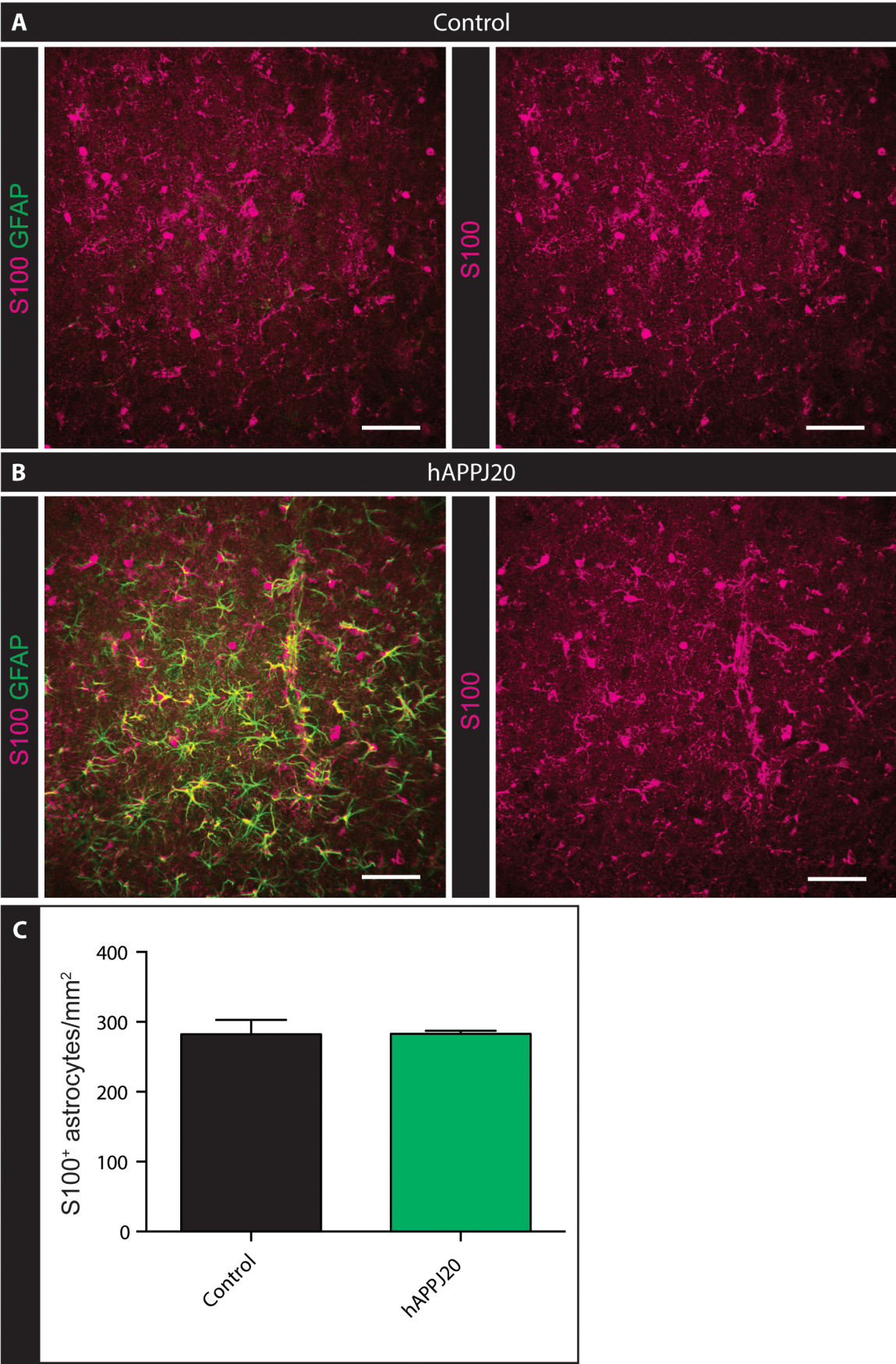

Supplement: Supplementary Table 1 [file suppl_data.zip › brain-2015-00380-File012.pdf]

Supplementary Figure 8 Baseline vessel diameters were unchanged in hAPPJ20 mice

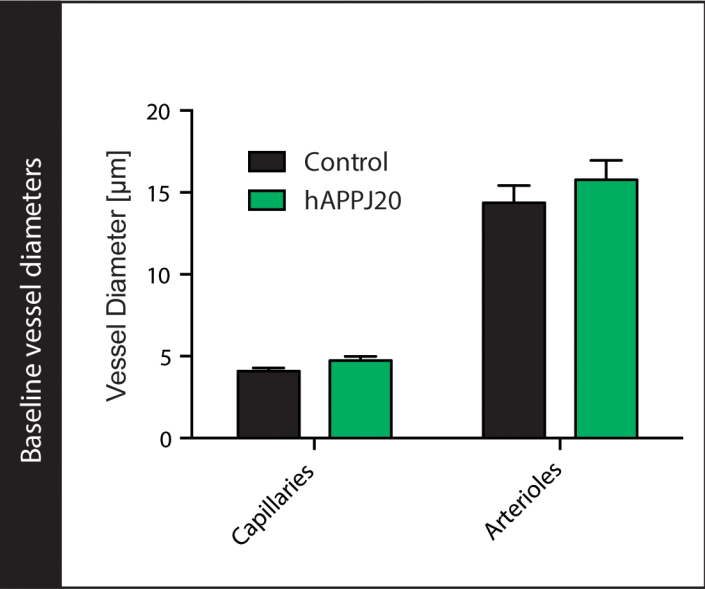

Supplement: Supplementary Table 1 [file suppl_data.zip › brain-2015-00380-File015.pdf]

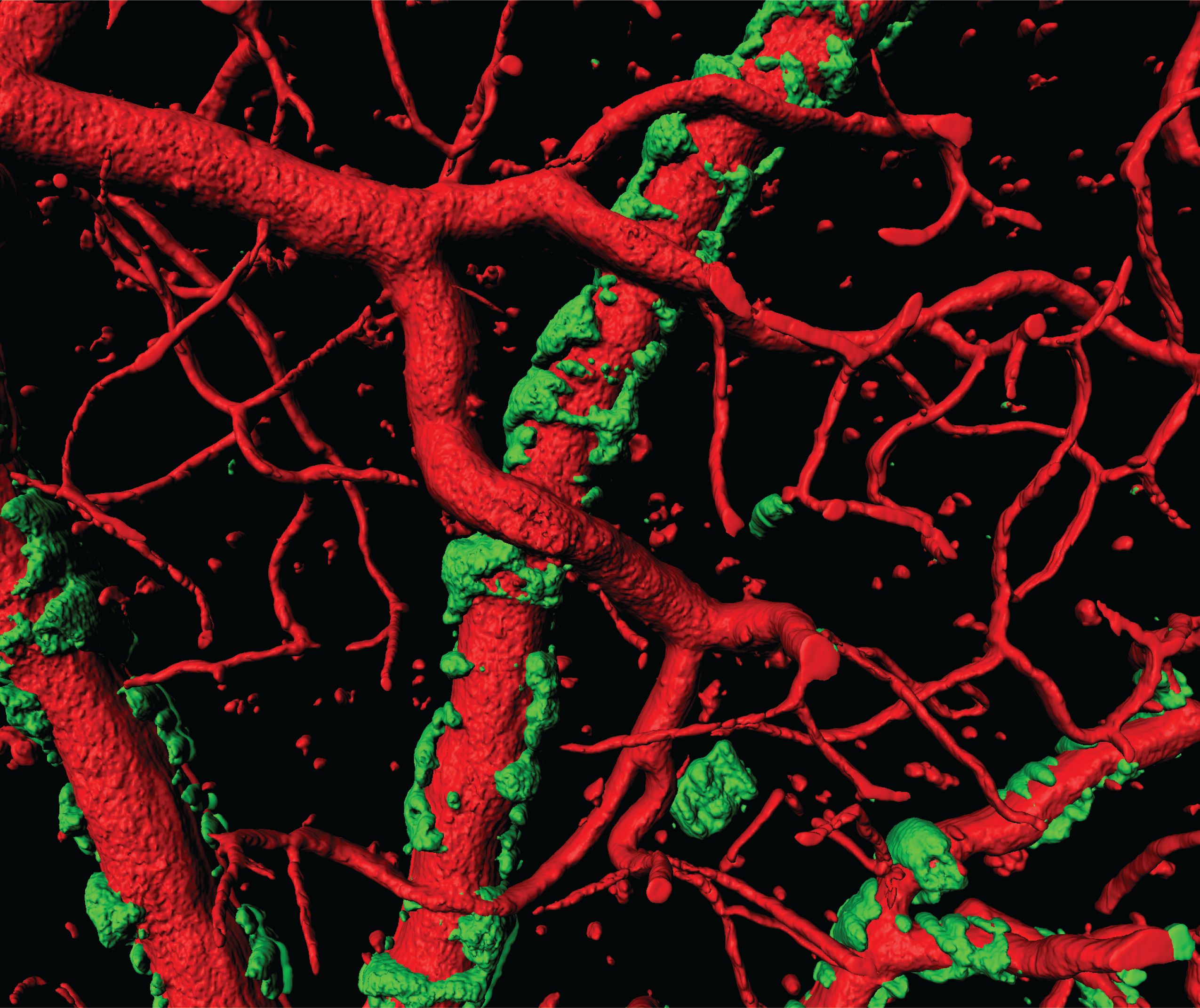

Supplement: Supplementary Table 1 [file suppl_data.zip › brain-2015-00380-File025.jpg]

Supplementary Figure 1 Calcium and vessel response in acute slices over time

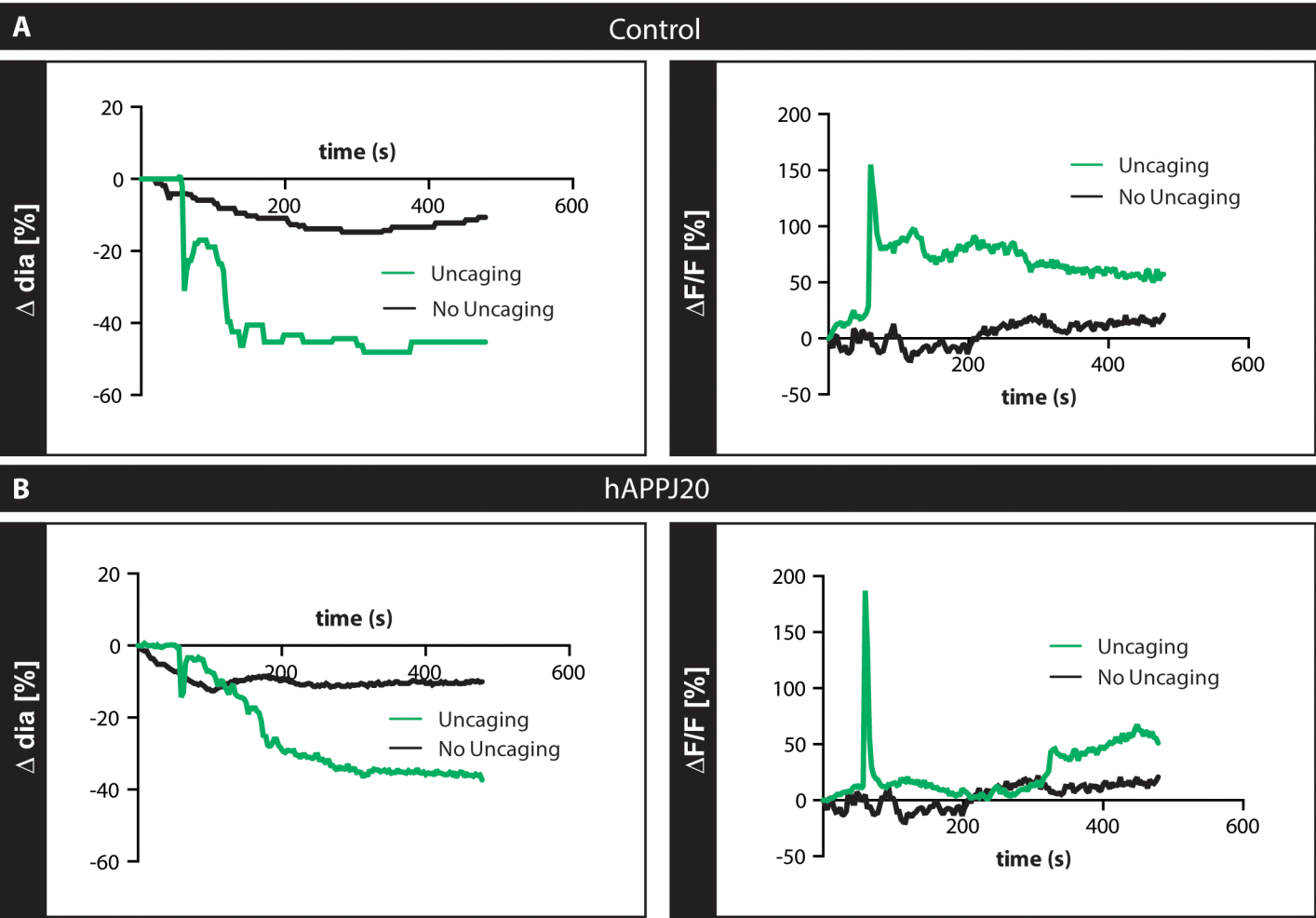

Supplement: Supplementary Table 1 [file suppl_data.zip › brain-2015-00380-File008.pdf]

Supplementary Figure 3 Aquaporin-4 is downregulated in astrocyte endfeet of hAPPJ20 mice

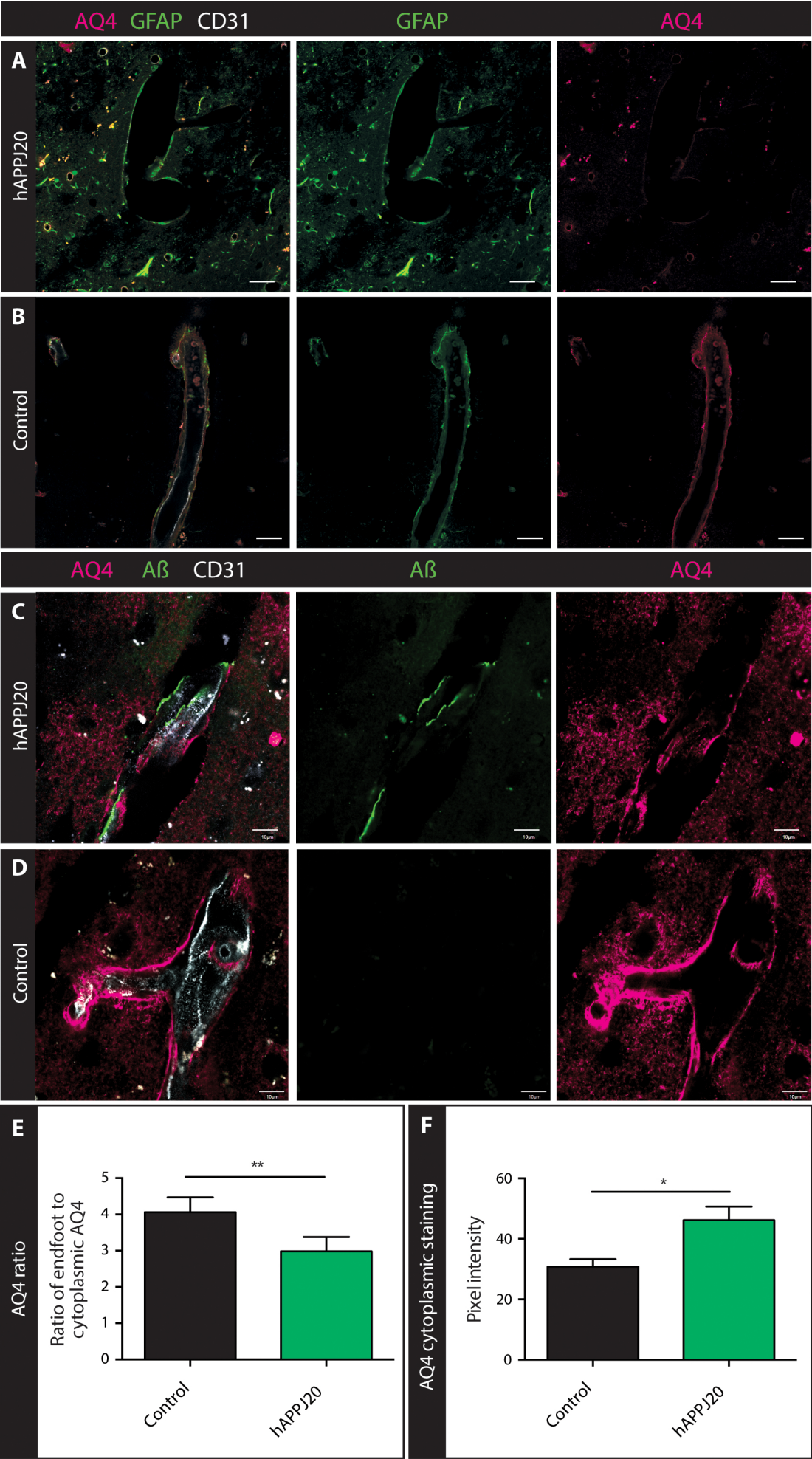

Supplement: Supplementary Table 1 [file suppl_data.zip › brain-2015-00380-File010.pdf]
